# The balance between the intronic miR-342 and its host gene Evl determines hematopoietic cell fate decision

**DOI:** 10.1038/s41375-021-01267-5

**Published:** 2021-05-21

**Authors:** Friederike Herbst, Tonio J. L. Lang, Elias S. P. Eckert, Peer Wünsche, Alexander A. Wurm, Tim Kindinger, Karin Laaber, Shayda Hemmati, Agnes Hotz-Wagenblatt, Oksana Zavidij, Anna Paruzynski, Junyan Lu, Christof von Kalle, Thorsten Zenz, Christoph Klein, Manfred Schmidt, Claudia R. Ball, Hanno Glimm

**Affiliations:** 1 Translational Functional Cancer Genomics, National Center for Tumor Diseases (NCT) and German Cancer Research Center (DKFZ), 69120 Heidelberg, Germany; 2 Charité - Universitätsmedizin Berlin, corporate member of Freie Universität Berlin and Humboldt-Universität zu Berlin, Department of Hematology, Oncology and Tumorimmunology, Augustenburger Platz 1, 13353 Berlin, Germany; 3 Faculty of Biosciences, University Heidelberg, 69120 Heidelberg, Germany; 4 Mildred Scheel Early Career Center, National Center for Tumor Diseases Dresden (NCT/UCC), Medical Faculty and University Hospital Carl Gustav Carus, Technische Universität Dresden, 01307 Dresden, Germany; 5 Department of Translational Medical Oncology, National Center for Tumor Diseases (NCT) Dresden and German Cancer Research Center (DKFZ), 01307 Dresden, Germany; 6 Center for Personalized Oncology, National Center for Tumor Diseases (NCT) Dresden and University Hospital Carl Gustav Carus Dresden at TU Dresden, 01307 Dresden, Germany; 7 Omics IT and Data Management Core Facility, German Cancer Research Center (DKFZ), 69120 Heidelberg, Germany; 8 BioNTech Manufacturing GmbH, 55131 Mainz, Germany; 9 European Molecular Biology Laboratory (EMBL), Genome Biology Unit, 69117 Heidelberg, Germany; 10 Department of Translational Oncology, National Center for Tumor Diseases (NCT) and German Cancer Research Center (DKFZ), 69120 Heidelberg, Germany; 11 GeneWerk GmbH, 69120 Heidelberg, Germany; 12 German Cancer Consortium (DKTK), 69120 Heidelberg, Germany; 13 Department of Medical Oncology and Haematology, University Hospital Zurich & University of Zurich, 8091 Zurich, Switzerland; 14 Department of Pediatrics, Dr. von Hauner Children’s Hospital, University Hospital, LMU Munich, 80337 Munich, Germany; 15 German Cancer Consortium (DKTK), 01307 Dresden, Germany

**Keywords:** Stem-cell research, Haematopoietic stem cells, Haematopoiesis

## Abstract

Protein-coding and non-coding genes like miRNAs tightly control hematopoietic differentiation programs. Although miRNAs are frequently located within introns of protein-coding genes, the molecular interplay between intronic miRNAs and their host genes is unclear. By genomic integration site mapping of gamma-retroviral vectors in genetically corrected peripheral blood from gene therapy patients, we identified the *EVL/MIR342* gene locus as a hotspot for therapeutic vector insertions indicating its accessibility and expression in human hematopoietic stem and progenitor cells. We therefore asked if and how EVL and its intronic miRNA-342 regulate hematopoiesis. Here we demonstrate that overexpression (OE) of Evl in murine primary Lin^−^ Sca1^+^ cKit^+^ cells drives lymphopoiesis whereas miR-342 OE increases myeloid colony formation in vitro and in vivo, going along with a profound upregulation of canonical pathways essential for B-cell development or myelopoietic functions upon Evl or miR-342 OE, respectively. Strikingly, miR-342 counteracts its host gene by targeting lymphoid signaling pathways, resulting in reduced pre-B-cell output. Moreover, EVL overexpression is associated with lymphoid leukemia in patients. In summary, our data show that one common gene locus regulates distinct hematopoietic differentiation programs depending on the gene product expressed, and that the balance between both may determine hematopoietic cell fate decision.

## Introduction

Hematopoiesis generates differentiated blood cells from stem and progenitor cells tightly controlled and adjusted to the needs of the organism by cell intrinsic and extrinsic factors [1–3]. Robustness of lineage differentiation is supported by microRNAs (miRNA) [4] on a posttranscriptional level. These small non-coding RNAs are either intronic or intergenic and mainly transcribed by Pol II leading to 21–24-bp double-stranded RNAs after several cleavage steps [5]. Knockout experiments of the miRNA-processing enzymes DGCR8 or DICER in distinct blood populations [6–8] clearly established that miRNAs are crucial for hematopoietic lineage determination.

Up to now, several miRNAs have been identified to play a role in hematopoietic lineage specification [9, 10] and stem cell self-renewal [11–13]. As an example, the knockdown of miR-126 in hematopoietic stem cells (HSCs) and early progenitors induces the expansion of the HSC pool without exhaustion by attenuating extrinsic signals [14]. Although more than 50% of annotated vertebrate miRNAs are intronic and more than two-thirds are co-expressed with their host genes [15], their relationship is not well understood as most studies on miRNA regulation do not address the interplay with their co-regulated genes.

Mechanistically, miRNAs and host genes can interact synergistically or antagonistically [16]. Synergistic functions have been described in cholesterol homeostasis, where miR-33 cooperates with the *Sterol Regulatory Element-Binding Protein* [17, 18], and neurogenesis, during which miR-338 targets genes that are antagonistic to its host the *Apoptosis Associated Tyrosine Kinase* [19]. By contrast, miR-26b functions in an antagonistic way by directly suppressing the *CTD Small Phosphatase 2* [20, 21]. Nevertheless, very little is known about the molecular interaction between a host gene and its miRNA in hematopoiesis.

We and others have previously shown that integration sites (IS) of gamma-retroviral vectors (γRV) into the host cell genome can be used to identify open and active chromatin regions within target cells [22–24]. By systematically analyzing the entire IS repertoire of therapeutic γRV in ten patients enrolled in the first German Wiskott–Aldrich syndrome (WAS) gene therapy trial we observed that clusters of IS mark hematopoietic regulatory genes, which are active in human bona fide HSCs. Notably, our study supports the application of γRV derived IS as neutral tags for cell-type-specific regulatory regions and that only a very limited number of γRV marked loci drive clonal selection [25]. Interestingly, we identified a significant cluster of IS close to the *ENAH-VASP-like* (*EVL*)/*MIR342* gene locus (among the top 50 tagged gene regions) after thoroughly investigating the IS pattern in peripheral blood cells from nine out of ten efficiently engrafted WAS patients [22, 25]. The continuous clonal contribution of cells harboring *EVL/MIR342* vector IS to blood formation for about five years points to a functional role of this genetic region in hematopoiesis. The *EVL* gene locus has mainly been reported to be involved in filopodia and lamellipodia formation in epithelial cells [26, 27] but has not been linked to hematopoiesis so far. Recently, it has been shown that miR-342 plays a role in the differentiation within the brain [28], but the function of EVL in this context was not investigated. To address the role of this common genomic locus encoding for a protein as well as a miRNA in hematopoiesis, we examined the individual and combinatory influence of EVL and its intronic miR-342 on blood cell differentiation.

## Methods

### Generation of lentiviral vectors

The genetic region of 266 bp harboring the *MIR342* locus was amplified from DNA extracted from the murine cell line 32D (for primer sequences see: Supplementary Table S1), subcloned and inserted into the 9928 bp plasmid pFCW.SIN.PPT.UbiC.mCherry.wPRE (LV.mCherry) using the restriction enzymes NheI and AscI, and verified by sequencing.

The codon-optimized coding sequence of the murine *Evl* gene (NCBI: NM_016337) was synthesized (ThermoFisher Scientific, Invitrogen GeneArt Gene Synthesis, Darmstadt, Germany) and inserted into the 8492 bp plasmid pCCL.SIN.cPPT.PGK.IRES.eGFP.wPRE (LV.eGFP) using the restriction enzymes SbfI and AsiSI, and verified by sequencing. VSV.G-pseudotyped concentrated lentiviral vector stocks for LV.eGFP, LV.Evl, LV.mCherry, and LV.miR-342 were produced by transient co-transfection as described in Herbst et al. [29], with minor modifications. In brief, the co-transfection of lentiviral packaging and transfer vectors was done by complex formation with polyethylenimine (PEI; Sigma, Deisenhofen, Germany) using a DNA:PEI ratio of 1:3.

### Quantitative real-time PCR analysis and global gene expression profiling

Total RNA was isolated using the RNeasy Kit (Qiagen, Hilden, Germany) for cell culture cells, or the ARCTURUS^®^ PicoPure^®^ RNA Isolation Kit (ThermoFisher Scientific, Applied Biosystems, Darmstadt, Germany) for murine hematopoietic primary cells. For quantitative real-time PCR, total RNA was transcribed in reverse using the Superscript III First-Strand Synthesis System (Invitrogen, Karlsruhe, Germany), and amplified using the Taqman^®^ Assay (ThermoFisher Scientific, Dreieich, Germany) or SYBR Green (LC480, Roche Diagnostics, Mannheim, Germany), for primer sequences see Supplementary Tables S2 and S3. The miRNeasy Mini Kit (Qiagen, Hilden, Germany) was used to extract and transcribe miRNA, which were examined by qRT-PCR using the miScript SYBR^®^ Green PCR Kit (Table S3). Signal intensities were normalized against the controls (Rnu6b or Tbp). Eleven days after lentiviral transduction, murine primary Lin^−^ Sca1^+^ cKit^+^ bone marrow (BM) cells were harvested and the RNA was extracted. Global gene expression profiling was conducted (*n* = 2 biological replicates per group) with the Illumina MouseWG-6v2_BeadChip system at the DKFZ Genomics and Proteomics Core Facility. For further details, see Supplementary Methods. The data discussed in this publication are accessible through GEO Series accession number GSE109600.

### Cell culture, lentiviral transduction

32D cells were cultured in RPMI-1640 (Invitrogen, Darmstadt, Germany) supplemented with 10% fetal bovine serum (PAN-Biotech, Aidenbach, Germany), 10% conditioned WEHI-medium, 2 mM l-glutamine (Invitrogen, Darmstadt, Germany) and 1% penicillin/streptomycin (GibCo, ThermoFisher Scientific, Dreieich, Germany). 293T cells were cultured in Iscove’s Modified Dulbecco’s Media (Invitrogen, Darmstadt, Germany) supplemented with 10% FBS, 2 mM l-glutamine, and 1% penicillin/streptomycin. J77A.1 cells were cultured in DMEM (4.5 g glucose; Invitrogen, Darmstadt, Germany) supplemented with 10% FBS, 2 mM l-glutamine, and sodium pyruvate. Jurkat cells were cultured in RPMI-1640 supplemented with 10% FBS and 2 mM l-glutamine. U-2 OS cells were cultured in McCoy’s 5A (GibCo, ThermoFisher Scientific, Dreieich, Germany) supplemented with 10% FBS and 2 mM l-glutamine.

Lin^−^ Sca1^+^ cKit^+^(LSK) cells were cultured in StemSpan^TM^ SFEM (StemCell Technologies, Cologne, Germany) supplemented with 100 ng/ml rmSCF, 100 ng/ml rmFlt3 Ligand, 100 ng/ml rhTPO, 20 ng/ml rmIL3 (all R&D Systems, Wiesbaden, Germany) and 1% penicillin/streptomycin. Cell lines were purchased from DSMZ, were authenticated using Multiplex Cell Authentication by Multiplexion (Heidelberg, Germany) as described recently [30], and are routinely tested for mycoplasma contaminations. The lentiviral transduction of 32D, 293T, J774A.1, Jurkat, U-2 OS cells or LSK cells was performed in the presence of Polybrene or Protaminesulfat (8 µg/ml; Sigma, Darmstadt, Germany) using a multiplicity of infection of 1–50.

### Western blot, luciferase assay, and CFC differentiation assay

Whole-cell extracts were obtained by using a cell lysis buffer (50 mM Tris-HCl (pH 7.4), 150 mM NaCl, 1% NP-40, 0.5% Na-deoxycholate, 0.1% SDS, protease inhibitor cocktail). After centrifugation at 13,000 rpm for 20 min, protein lysates were loaded onto SDS-PAGE and transferred to a PVDF membrane (Invitrogen, Darmstadt, Germany). Antibodies are listed in Supplementary Table S4.

For luciferase assays, 1–5 × 10^4^ 293T cells either transfected with 0.5 µg miR-342 expression construct, 0.02 ng miR-342-3p or miR-342-5p mimics (miRIDIAN, Horizon Discovery, Cambridge, UK) or 293T cells ectopically expressing miRNA or control vector were used. 293T cells were further transfected using PEI with 0.05 or 0.25 µg psiCheck2 vectors (Promega, Walldorf, Germany), respectively containing the target gene-specific 3′UTR- or miR-342-binding sites of the sponge vector (cloning primers in Table S1). After 48–72 h of transfection, the luciferase activity was measured using the Dual-Luciferase^®^ Reporter Assay System Kit (Promega, Walldorf, Germany) according to the manufacturer’s protocol. All experiments were performed in biological replicates (*n* = 3) using technical replicates (*n* = 3). Investigators were blinded to group allocation until the evaluation of data followed by interpretation.

For myeloid colony-forming unit (CFU) assays, 4000 fluorescence-positive LSK cells were plated using MethoCult™ GF M3434 (StemCell Technologies, Cologne, Germany). For lymphoid CFU assays, 8000 sorted LSK cells were plated using Methocult^TM^ M3630. Colonies were quantified 10 days after incubation at 37 °C and 5% CO_2_. All CFU assays were performed in biological replicates (*n* = 3) using technical replicates (*n* = 2).

### AGO2 co-immunoprecipitation, low RNA high-throughput sequencing, and bioinformatical analysis

A total of 2 × 10^7^ cells per sample were lysed with an ice-cold polysome buffer (10 mM HEPES (pH 7), 100 mM KCl, 5 mM MgCl_2_, 0.5% NP-40, 10 mM DTT, 2.25 U RNaseOut, protease inhibitor cocktail, 0.4 mM Vanadyl ribonucleoside complex), and stored overnight at −80 °C. Immunoprecipitation was performed with 5 µg of anti-Ago2 antibody, or the mouse IgG isotype control coupled with Dynabeads (ThermoFisher Scientific, Dreieich, Germany) for 4 h at 4 °C in NT2 buffer (50 mM Tris (pH 7.4), 150 mM NaCl, 1 mM MgCl_2_, 0.05% NP-40, 0.5 U RNaseOut, 0.4 mM Vanadyl ribonucleoside complex, 1 mM DTT, 0.015 mM EDTA (pH 8), protease inhibitor). After washing the beads three times with ice-cold NT2 buffer, the proteins were eluted by incubating the samples with Glycin (pH 2.3) for 15 min followed by neutralization with Tris-HCl (pH 8). Proteins were digested using Proteinase K and removed by QIAzol/Chloroform extraction. RNA was isolated using the miRNeasy Kit according to the manufacturer’s protocol.

Total RNA fractions (*n* = 2 technical replicates/group) were submitted for low RNA HTS using Illumina HiSeq2000 (paired end, 125 bp). For further details see Supplementary Methods. The common predicted miR-342 target genes (from miRWalk, miRanda, RNA22, and Targetscan) were downloaded from miRWalk2.0 and used in BioVenn to identify the intersection between genes expressed in LSK cells (RPKM > 800) and the enriched genes after AGO2 pulldown.

### Flow cytometry

Cells (for details about the immunophenotype of enriched cell fractions see Table S5) were dissociated, washed once with Hanks' balanced salt solution (HBSS; Sigma, Darmstadt, Germany) containing 2% FBS, and analyzed for eGFP or mCherry positivity by flow cytometry (AriaII, LSRII, FACS-Fortessa; Becton Dickinson, Heidelberg, Germany).

The peripheral blood from the recipient mice was collected monthly. After lysis of erythrocytes (0.15 M ammonium chloride), the cells were washed with HBSS containing 2% FBS, stained with Fluoro-Gold (ThermoFisher Scientific, Dreieich, Germany) for dead cell exclusion and with fluorochrome-conjugated monoclonal antibodies (Table S6), for further analysis by FACS for eGFP or mCherry expression in addition to lineage markers. Spleens were harvested, cells were singularized using a cell strainer (40 µm, Falcon^®^), washed and stained for hematopoietic lineage markers.

### Bone marrow transplantation and colony-forming unit-spleen

BM cells were harvested from the hind legs and spines of 6–8-week-old male B6.SJL-Ptprc^a^Pepc^b^/BoyJ (Ly 5.1) mice, transduced with LV, and injected intravenously into lethally irradiated (950 cGy) female C57Bl6/J (Ly 5.2) mice [29]. In brief, lineage depletion of the harvested BM was performed using EasySep (StemCell Technologies, Cologne, Germany), further enriched for LSK markers (Table S6) by FACS, and transduced overnight with LV. After 36 h, cells were injected intravenously into lethally irradiated recipients (2500 LSK cells per mouse, *n* = 6 per group). BM and spleens were harvested after 4 months and 2 × 10^7^ total BM cells/mouse were re-transplanted into secondary recipients (*n* = 6/group).

For CFU-Spleen limiting-dilution assays, 530, 1440, or 14,400 cells per mouse were injected intravenously into lethally irradiated recipients (1050 cGy). After 13 days of transplantation, spleens were harvested, weighed, and colonies were counted. Investigators were blinded to group allocation until mice were sacrificed. L-Calc Software was used to determine the frequency of CFU-S. 5 (+1 substitute) mice were used per group as explorative cohort and randomly allocated into experimental groups. Mice were housed under pathogen-free conditions and all experiments were conducted according to the German animal protection laws and regulations approved by the ethical committee.

### Statistical analysis

Results are presented as mean ± s.d. if not indicated differently. Statistical significance was determined using the Student’s *t*-test. Biological triplicates and technical replicates were used, if not indicated differently.

## Results

### IS-tagged *EVL/MIR342* hematopoietic clones are long-term active in human hematopoiesis

We identified the *EVL/MIR342* locus by subsequently analyzing the whole integrome of ten patients treated with clinical gene therapy for the correction of WAS for up to 6 years post transplantation [22, 25]. WAS is an X-linked, primary immunodeficiency disorder caused by mutations in the *WAS* gene. Patients suffer from recurrent infections, thrombocytopenia, and autoimmunity, and have an increased risk of lymphoma development [31]. The mutated WAS protein leads to dysfunctional leukocytes including lymphoid and myeloid cells [32]. Treatment options consist of allogeneic HSC transplantation or gene therapy approaches using patient-derived CD34^+^ cells for genetic modification. Here, we discuss data from the German WAS gene therapy trial [22] using γRV gene delivery vehicles.

Hematopoietic clones carrying therapeutic vector integrations near the *EVL/MIR342* locus were detected in peripheral blood and BM samples of all patients post-transplant and this locus was among the top 50 enriched regions marked by common integration sites (CIS). Recently, we showed that the number of CIS in hematopoietic clones of WAS patients strongly correlates with chromatin accessibility in HSCs but not with the level of gene expression of the IS-tagged loci [25]. As gamma-retrovirus derived vectors can serve as neutral molecular tags by preferentially integrating into cell-type-specific active and open chromatin regions [24, 33, 34], we hypothesized that the *EVL* and *MIR342* genes are expressed and functionally relevant in normal human long-term hematopoiesis. Moreover, clonal outgrowth due to insertional mutagenesis is restricted to a very defined and limited number of clones and there is no known function of *EVL* and *MIR342* genes in WAS.

**Fig. 1:**
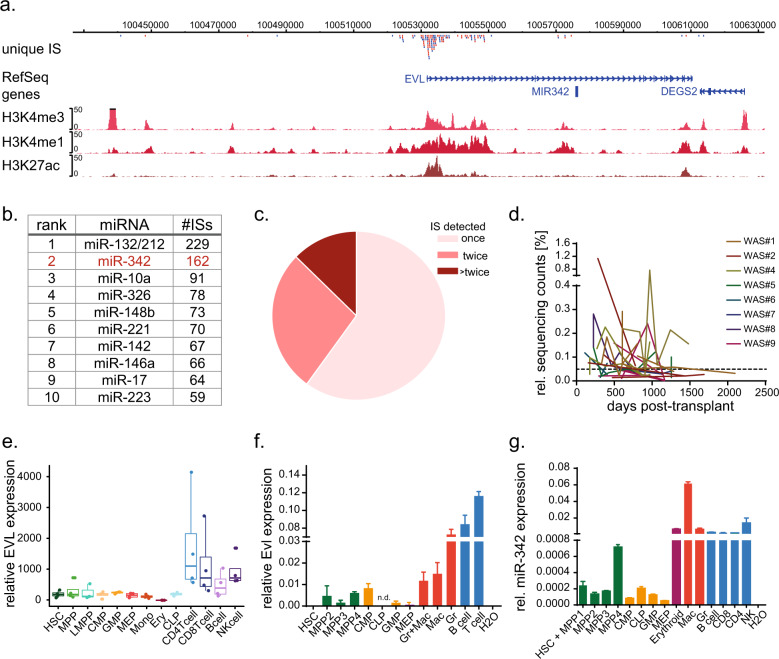
Identification of the *EVL/MIR342* gene locus as a novel candidate regulatory region in hematopoiesis. **a** Genomic localization of all therapeutic vector integration sites (IS) close to the *EVL/MIR342* gene locus detected in ten Wiskott–Aldrich syndrome gene therapy patients. Histone modifications in human CD34^+^ cells representing promoter (H3K4me3), enhancer (H3K4me1) and active (H3K27ac) chromatin regions. H histone, K Lysine, me methylated, ac acetylated. **b** Top-ranked miRNAs according to the number of therapeutic vector integration sites (ISs) nearby in efficiently engrafted Wiskott–Aldrich syndrome patients (*n* = 9). **c** Percentage of IS, which were sequenced once (light red), more than once (red) and more than twice (dark red) in WAS gene therapy patients during the cause of the study (2097 days post-transplant). **d** Clonal dynamics of individual gene-corrected cells harboring IS close to the *EVL/MIR342* locus, which were repeatedly (>twice, Fig. 1b) detected in WAS patients for up to 2097 days post-transplant. Dashed line represents the median of all repeatedly sequenced IS. **e** RNA-seq counts for EVL transcripts in defined human hematopoietic cell populations. **f** Relative expression of Evl (normalized to Tbp) and **g** miR-342-3p (normalized to Rnu6b) in enriched murine hematopoietic primary populations. Indicated cell populations were isolated from 8- to 10-week-old C57/Bl6 mice using immunophenotypic markers, RNA was isolated and subjected to qRT-PCR. HSC hematopoietic stem cell, MPP multipotent progenitor, CMP common myeloid progenitor, CLP common lymphoid progenitor, GMP granulocyte macrophage progenitor, MEP megakaryocytic erythroide progenitor, Gr granulocyte, Mac macrophage, CD8 T cell, CD4 T cell, NK1.1 natural killer cell, n.d. no data.

First, we analyzed the genomic elements close to the 165 insertions derived from unique clones (identified in all patients analyzed) in vicinity to the *EVL/MIR342* locus. Vector integrations within patient-derived cells clustered close to the transcription start site (TSS) of *EVL* (+44.4 to −75.5 kb) (Fig. 1a). Next, we applied the same selection criteria to conserved miRNA genes only and identified miR-342 as the second top-ranked miRNA following miR-132/212 (Fig. 1b), a known regulator of HSC maintenance [13]. Eight out of the top ten-ranked miRNAs have been proven to play a role in hematopoiesis. Furthermore, 40% of the unique clones were detected more than once within individual patients (Fig. 1c) and 21 clones out of those were sequenced several times for the observation period of more than 5 years in hematopoietic samples clearly showing their long-term hematopoietic activity (Fig. 1d).

### Evl and miR-342 are co-expressed in murine hematopoietic cells

We analyzed the expression of EVL in human blood cell populations starting from HSCs via progenitors to differentiated cells (Fig. 1e) using publicly available datasets from Corces et al. (10.1038/ng.3646) and next verified the expression level of Evl and miR-342-3p in defined murine hematopoietic cell populations (Fig. 1f, g). It is important to note that the conservation of the EVL protein is 94% between human and mouse (Supplementary Fig. S1a) and that both species share 100% identical 5p and 3p mature miR-342 sequences.

Both genes were expressed at low level in murine HSC and progenitor cells (MPP1-4), which is in line with published whole-transcriptome sequencing data [35], and their expression increased profoundly in differentiated blood cells (Fig. 1f, g). The highest expression of Evl was detected in lymphocytes (13.8 ± 0.3–19.1 ± 0.9-fold higher compared to MPP4 cells), whereas miR-342 was expressed at the highest level in macrophages (85.8 ± 0.4-fold higher compared to MPP4 cells).

However, we observed co-expression of both gene products in murine hematopoietic primary cells (Fig. 1f, g) as well as in murine and human cell lines (Supplementary Fig. S1b, c). This suggests that the intronic miR-342 is co-regulated with its host gene *Evl*, which is in line with its previous classification as a canonical and host gene co-expressed miRNA [16, 28]. To gain insights into the regulatory landscape and chromatin structure of the locus in human hematopoietic cells, we analyzed a recently published data set from Corces et al. defining open chromatin by ATAC-Seq (Assay for Transposase Accessible Chromatin with high-throughput sequencing) in human CD34^+^ cells and physical genetic interaction determined by promoter capture HiC sequencing published by Mifsud et al. (10.1038/ng.3286). We identified enhancer marks (H3K4me1) upstream of both, the *EVL* TSS and the *MIR342* gene locus (Fig. 1a). Yet, acetylated H3K27 indicating active chromatin was only detected upstream of *EVL*. Genomic interaction was indicated between the *MIR342* region and the promoter/enhancer sequences in front of the *EVL* TSS (Fig. S2), and myeloid transcription factor binding sites (data not shown) were present close to *MIR342*, which is in accordance to its highest expression in murine myeloid cells (Fig. 1g). Noteworthy, the chromatin accessibility at the *MIR342* locus was lowest in human common lymphoid progenitors (CLPs) and B-cells compared to all other cell types (Fig. S2).

### Overexpression of EVL drives lymphopoiesis whereas miR-342 promotes myeloid differentiation

**Fig. 2:**
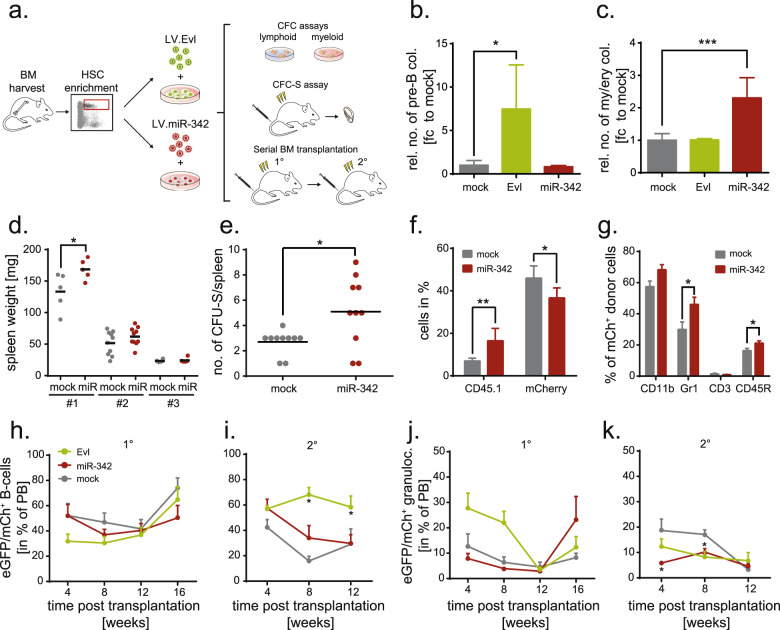
EVL drives lymphopoiesis whereas miR-342 promotes myeloid differentiation in vitro and in vivo. **a** Experimental setup scheme. Bone marrow (BM) was harvested from Bl6 mice, enriched for Lin^−^ Sca1^+^ cKit^+^ (LSK) cell markers by flow cytometry and cells were transduced with overexpression (OE) lentiviral vectors for Evl or miR-342 (and mock as a control). The influence of Evl and miR-342 OE on the hematopoietic differentiation capacity was assessed by lymphoid and myeloid colony-forming assays (CFC) in vitro or a CFC-Spleen (S) assay in vivo. Multilineage reconstitution and self-renewal were addressed by serial BM transplantation. **b** Relative number of colonies derived from EVL-, miR-342- or mock overexpressing LSK cells in pre-B-cell growth supporting semisolid medium (*n* = 3). **c** Relative number of colonies derived from EVL-, miR-342- or mock overexpressing LSK cells in erythroid and myeloid growth supporting semisolid medium (*n* = 3). **d** Weight of spleens in mg of mice transplanted with 14,400 LSK cells/mouse (group #1), 1440 LSK cells/mouse (group #2), and 530 LSK cells/mouse (group #3) transduced with miR-342 or mock overexpression vectors 13 days post-transplant. **e** Number of myelopoietic progenitor-derived colony-forming units (CFU) within the spleens of mice transplanted with LSK cells OE miR-342 or mCherry control 13 days post-transplant. **f** Frequency of donor-derived (CD45.1) and transduced (mCherry) cells within spleens of mice at day 13 after transplantation of miR-342 OE or mock LSK cells. **g** Frequency of myeloid (CD11b, Gr1) and lymphoid (CD3, CD45R) hematopoietic cells within the mCherry-positive donor cell fraction in spleens of mice at day 13 after transplantation of miR-342 OE or mock LSK cells. **h** Frequency of donor-derived eGFP/mCherry-positive peripheral B-cells in primary (1°) and **i** secondary (2°) recipient mice. **j** Frequency of donor-derived eGFP/mCherry-positive peripheral granulocytes in primary (1°) and **k** secondary (2°) recipient mice. *P < 0.05; **P < 0.01; ***P < 0.001; **h**–k (*n* = 6 mice/group; SEM).

Firstly, we validated the stable lentiviral mediated overexpression (OE), which is comparable with physiologic levels detected in murine and human cell lines or hematopoietic primary cell populations (data not shown), and assessed the functionality of Evl and miR-342-3p in 32D and 293T cells (Fig. S3). In a next step, we addressed whether OE of either Evl or miR-342 affects the differentiation capacity of murine stem and progenitor LSK cells in vitro and in vivo (Fig. 2a). OE of Evl led to a 7.5 ± 5.1-fold increase of pre-B cell colonies compared to control vector-transduced LSK cells (Fig. 2b). By contrast, miR-342 overexpressing cells formed a 2.3 ± 0.6-fold higher number of myeloid colonies (Fig. 2c). To quantify the myeloid colony-forming potential of miR-342^+^ LSK cells in vivo, we analyzed myeloid spleen colonies after transplantation. In line with the in vitro data, we detected enlarged spleens (Fig. 2d; group #1, *P* = 0.047) and a 2.4 ± 0.6-fold increase in colonies (Fig. 2e; *P* = 0.0183) due to a higher number of miR-342-positive donor-derived cells (Fig. 2f, *P* = 0.007). In total, we assessed a frequency of 1/275 CFU-S in miR-342^+^ LSK cells compared to 1/486 CFU-S in control vector-transduced LSK cells determined by limiting dilution, as well as a higher proportion of donor-derived myeloid cells in the spleens (Fig. 2g; *P* = 0.042). Serial BM transplantation experiments demonstrated that both, EVL and miR-342 overexpressing cells, generated stable multilineage reconstitution (Fig. 2h–k). However, a 4.3-fold higher frequency of EVL-positive B-cells was detected in peripheral blood 4 and 8 weeks (*P* = 0.93e−6) after secondary transplantation (Fig. 2i). Moreover, EVL^+^ primary and secondary recipients harbored a higher donor-derived B-cell frequency within their spleen (1° spleen: *P* = 0.06 and 2° spleen: *P* = 0.07; Fig. S4) but donor-derived granulopoietic cell numbers were largely unaffected (Fig. 2k).

**Fig. 3:**
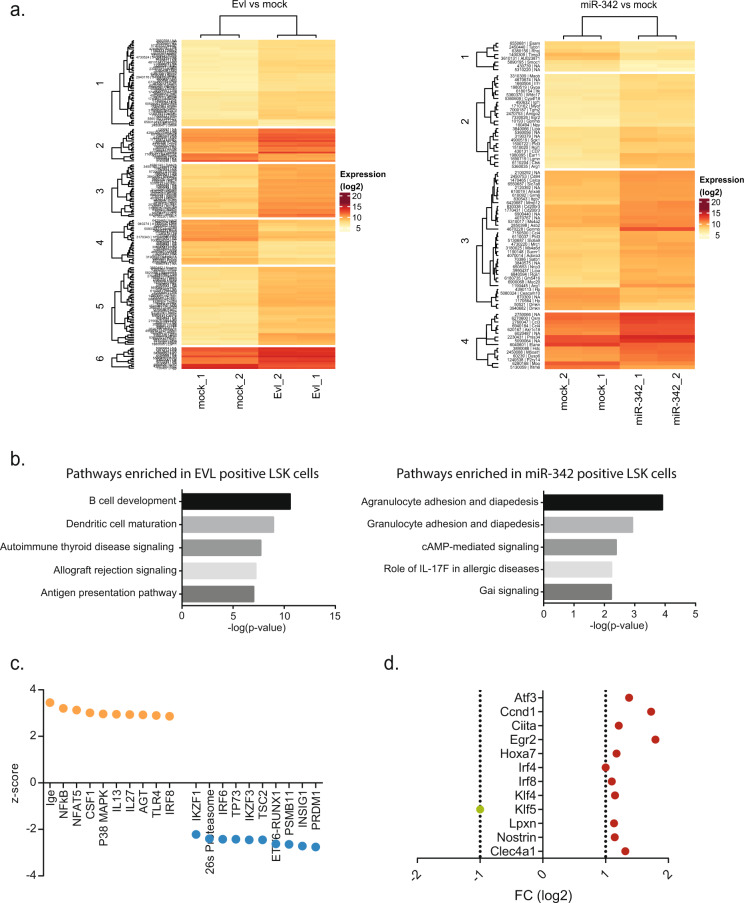
Overexpression of Evl deregulates pathways important to lymphoid cells whereas miR-342 alters myeloid associated signaling. **a** Global gene expression profiling of LSK cells overexpressing Evl or miR-342 after 11 days in culture. 190 and 90 transcripts were significantly deregulated (FoldChange > 2) in Evl or miR-342 overexpressing cells compared to mock transduced cells, respectively. **b** The top five deregulated pathways upon Evl or miR-342 overexpression are displayed ranked by their −log(*p* value). **c** Potential upstream regulators identified in primary LSK cells after overexpression of Evl. Orange circles indicate activation (positive z-score) in murine primary LSK cells upon Evl expression and blue circles represent potential inhibited regulators (negative z-score) based on microarray data analysis using the Ingenuity Pathway Analyzer in Evl expressing (Evl OE) and mock transduced control (ctrl) LSK cells. **d** FC in log2 of the 12 transcription factors deregulated in Evl OE LSK cells compared to mock ctrl.

To gain further insights into the molecular function of EVL and miR-342, we performed global gene expression profiling of transduced LSK cells. We observed 191 and 85 significantly differentially expressed genes upon Evl or miR-342 OE, respectively (Fig. 3a). Interestingly, 32% (62 out of 191) of the deregulated transcripts were involved in hematopoietic system development and function after Evl OE. Moreover, the top deregulated canonical pathways detected are essential for the development of B-cells (*P* = 0.26e−12). By contrast, genes relevant to myeloid cell functions, such as granulocytic adhesion and diapedesis (*P* = 0.26e−4) were significantly upregulated within miR-342-positive LSK cells (Fig. 3b). Next, we used the upstream regulator analysis to identify the activity of gene regulatory networks, and to predict transcriptional regulators. The activation z-score was used to select the top ten activated and inhibited pathways upon Evl OE. Here, we revealed that apoptosis and cell death-associated pathways are inhibited, whereas lymphopoiesis, cell movement and invasion are activated (Fig. S3e). In particular, interferon and cytokine signaling, MHC class II antigen presentation and TCR activation are enriched indicating for pathways in developing lymphocytic cells (data not shown). Subsequently, we extracted the top 20 predicted cellular upstream regulators identified based on the Evl^+^ LSK cell transcriptome (Fig. 3c). As an example, we found TRAF6 mediated NFκB signaling activated in hematopoietic primary cells upon Evl OE (z-score 3.2; *p* value of overlap genes *P* = 0.28e−11, Fisher’s exact test). To identify drivers of lymphoid diversification, we selected for deregulated transcription factors in EVL^+^ LSK cells compared to mock transduced cells (Fig. 3d). *Egr2*, with the highest log2 FC of 1.8, has been shown to regulate Hox genes like *HoxA4*, important for HSC and proB cell expansion [36] or *HoxB3* blocking B cell progenitors, T cell differentiation or delaying myeloid precursor proliferation [37] when overexpressed which suggests for a role in balancing myeloid and lymphoid fractions. Thus, EVL is associated with drivers of lymphopoiesis, whereas miR-342 OE results in upregulation of myeloid-specific pathways.

### Antagonism of miR-342 and EVL in hematopoietic lineage differentiation

**Fig. 4:**
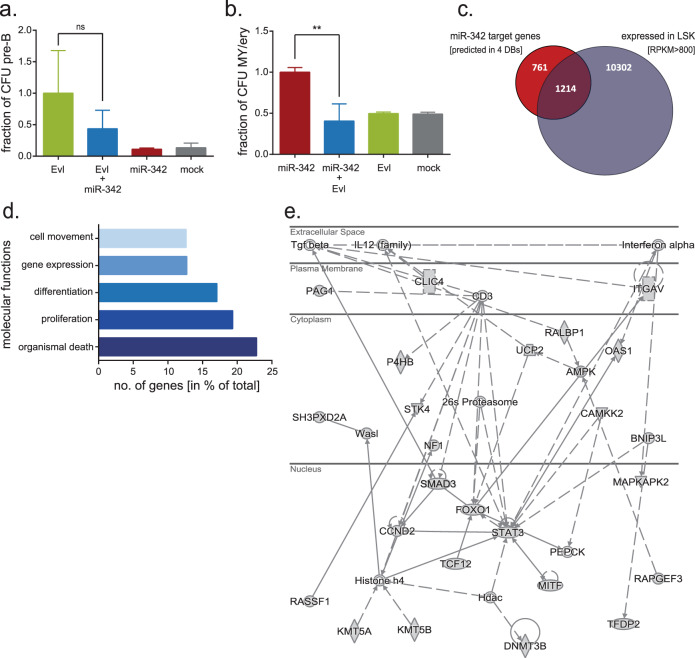
Co-expression of the host gene Evl and its intronic miR-342 neutralizes their individual hematopoietic differentiation potential. **a** Relative fraction of colony-forming units (CFU-pre B) derived from LSK cells in pre-B cell growth supporting semisolid medium upon Evl, Evl and miR-342, miR-342 or mock transduction. **b** Relative fraction of colony-forming units (CFU-GM) derived from LSK cells in myeloid and erythroid (My/ery) growth supporting semisolid medium upon miR-342, miR-342 and Evl, Evl or mock transduction. **c** Predicted miR-342 target genes identified in four different databases (DB: miRWalk, miRanda, RNA22, and TargetScan) and their expression on RNA level (RPKM > 800) in murine primary LSK cell populations [35]. **d** Pathway analysis using the 1214 in silico-derived miR-342 target genes (Fig. 4c) to describe their molecular functions ranked by the number (no.) of genes in % of total genes within the given category. **e** One of the top three networks (Score 30–36) targeted by miR-342 identified by in silico analysis (adapted from Ingenuity Pathway Analysis). All identified miR-342 targets within this network are displayed with gray shaded symbols. Solid lines represent direct and dashed lines indirect interactions between molecules based on the IPA database.

As miR-342 is co-expressed with its host gene *Evl*, we analyzed hematopoietic differentiation after simultaneous OE of both genes. Interestingly, miR-342 expression in EVL^+^ LSK cells reduced the lymphoid-primed differentiation capacity of EVL 0.43 ± 0.29-fold (Fig. 4a). Furthermore, co-expression of Evl and miR-342 abolished the myeloid-biased colony formation induced by miR-342 OE alone (Fig. 4b). In vivo, the frequency of donor-derived T-cells detected in the spleen of secondary mice was reduced upon miR-342 OE compared to Evl OE or controls (Fig. S4b).

**Fig. 5:**
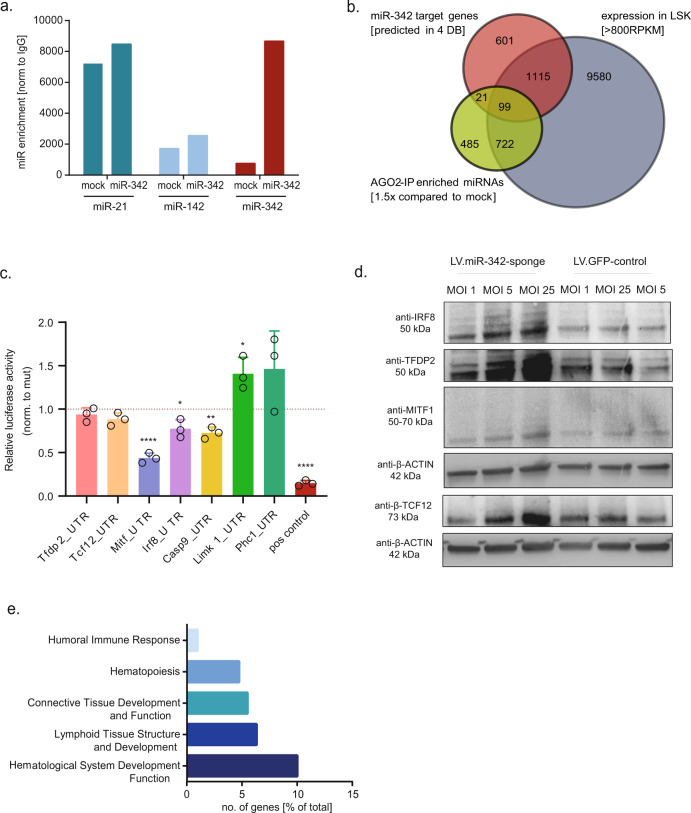
MiR-342 antagonizes EVL by targeting mRNAs, which are involved in lymphoid signaling pathways. **a** Relative enrichment of the indicated miRNAs (miR-21, miR-142, miR-342) in mock and miR-342 (miR) overexpressing hematopoietic cells after AGO2 immunoprecipitation compared to IgG control samples. **b** Venn diagram of experimentally identified miR-342 target mRNAs (AGO2-IP enriched mRNAs) compared to in silico predicted targets and mRNAs expressed in LSK cells (Fig. 4c). **c** Relative luciferase activity (normalized to individual mutated UTR vector-transfected cells) in 293T cells after transfection with specific 3′UTR sequence containing constructs of predicted miR-342 target genes and the miR-342 overexpression vector. *P < 0.05; **P < 0.01; ***P < 0.001. **d** Western blot analysis of identified miR-342 target genes with lysates of murine bone marrow-derived 32D cells after inhibition of miR-342 using increasing amounts (MOI 1, 5, 25) of a specific miR-342 sponge vector (LV.miR-342-sponge) or its corresponding GFP-expressing control vector (LV.GFP-control). **e** Functional classification of the mRNAs, which are 1.5-fold enriched in miR-342 overexpressing samples compared to mock cells after AGO2-IP ranked by the number (no.) of genes in % of total genes within the given category.

We performed in silico analysis to identify miR-342 targets (3p and 5p) and selected 1214 potential candidates (Fig. 4c). Strikingly, miR-342 predominantly targets pathways involved in cell movement (especially T-cell migration as major subcategory), differentiation and proliferation (main cell class affected: lymphocytes; not shown) (Fig. 4d). This suggests that miR-342 antagonizes its host gene’s function, which drives lymphopoiesis. It is important to note that the Evl mRNA does not contain any miR-342 binding sites and that we observed no significant influence on Evl mRNA levels upon ectopic miR-342 expression and vice versa (Fig. S5a, b), pointing towards an indirect and downstream mode of action. One of the top miR-342 inhibited networks in silico is downstream of CD3, which is part of the T-cell receptor (Fig. 4e). To confirm miR-342 target genes experimentally, we performed AGO2 co-immunoprecipitation and detected a strong enrichment (8674-fold compared to IgG) of the ectopic miR-342 in the RISC complex without replacing and unphysiologically outcompeting endogenously expressed miRNAs like miR-21 and miR-142 (Fig. 5a). High-throughput sequencing of AGO2 co-immunoprecipitated mRNA fractions revealed 1327 significantly enriched mRNAs (≥1.5-fold compared to AGO2-IP samples of mock cells), of which 73.32% were bound with their 3′UTR. Next, we identified 99 candidates whose mRNA expression in LSK cells overlaps with the prediction and experimental assessment as a miR-342 target (Fig. 5b; Table S7). To validate the experimental strategy, we selected seven genes with different mRNA enrichment scores (fold changes between 3.0 and 25.7) in the miR-342- versus control-immunoprecipitated AGO2-complexes. We assessed the miR-342 dependent repression of gene-specific 3′UTR containing luciferase constructs (Fig. 5c, Fig. S6) and showed that increasing inhibition of miR-342 leads to gradually elevated protein levels of four in silico predicted and AGO2-IP detected miR-342 targets (Fig. 5d), which are suggested to be involved in lymphoid signaling pathways (see Fig. 4e). Among the top canonical pathways suppressed by miR-342 are SAPK/JNK signaling and B-cell activating factor signaling, which belong to the molecular categories of hematological system development and function and lymphoid tissue structure and development (Fig. 5e).

### EVL is highly expressed in lymphoid leukemia

**Fig. 6:**
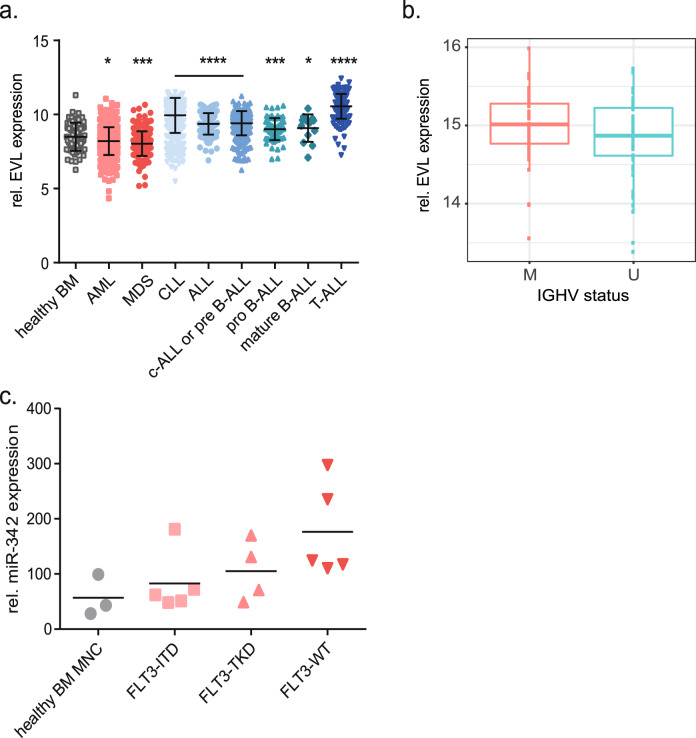
EVL is significantly overexpressed in lymphoid leukemia compared to healthy bone marrow and myeloid malignancies. **a** Expression of EVL in 2096 patient samples with hematopoietic malignancies compared to healthy bone marrow (BM). AML acute myeloid leukemia, MDS myelodysplastic syndrome, CLL chronic lymphoid leukemia, ALL acute lymphoid leukemia. **b** Association of the expression level of EVL with immunoglobulin heavy chain gene (IGHV) mutation status in 120 CLL samples. M mutated, U unmutated. **c** Relative miR-342-3p expression identified by small RNA-seq in the indicated AML samples with normal karyotype compared to healthy bone marrow-derived mononuclear cells (BM MNC). FLT3 FMS-related tyrosine kinase 3, ITD internal tandem duplications, TKD tyrosine kinase domain. *P < 0.05; **P < 0.01; ***P < 0.001.

The genomic *EVL/MIR342* locus was previously shown to be frequently methylated in colon cancer [38] and upregulated EVL expression was reported in breast cancer [39]. We then asked whether expression of EVL is associated with a specific subtype of leukemia. To address this, we re-analyzed the publicly available gene expression data set of 2096 leukemia samples of the MILE study (Microarray Innovations in LEukemia) from the ELN (European Leukemia Network). EVL was one of the top 100 differentially expressed genes between ALL and AML samples. Strikingly, we detected the highest expression of EVL in all subtypes of lymphoid leukemia, especially in T-ALL compared to healthy BM or myeloid malignancies (Fig. 6a). Within an independent data set of 120 CLL patients [40], EVL expression is associated with mutated IGHV (*P* = 0.037; Fig. 6b). To get further insights into miR-342 expression in myeloid leukemia, we analyzed a small RNA sequencing data set of AML patients, grouped based on the mutational status of the AML driver gene *FLT3*, in comparison to healthy controls (*n* = 17) [41]. We observed increased miR-342 expression in AML samples, especially in patients without a *FLT3* mutation, compared to healthy BM-derived mononuclear cells that did not reach statistical significance (*P* = 0.06; Fig. 6c). Analyzing the TCGA-LAML cohort of about 188 cases revealed that higher expression of mir-342 is associated with the morphological (FAB-) subtype M0 (*P* = 0.04) and the adverse risk category (according to ELN; *P* = 0.005; Table S8). Molecularly, this analysis indicates that high mir-342 expression correlates with mutations in *RUNX1* (*P* = 0.009), and *TP53* (*P* = 0.0006). In contrast, low mir-342 expression is significantly associated with mutated *NPM1* (*P* < 0.0001). These data suggest that the *EVL/MIR342* locus may play a role in leukemia, however its potential prognostic impact needs further investigation.

## Discussion

We here describe a genomic locus shared by a protein-coding gene and a miRNA that was identified by γRV tags in human repopulating long-term HSCs of ten clinical gene therapy patients. Our data demonstrate that the protein-coding gene *Evl* and its intronic miR-342 counterbalance each other in hematopoietic lineage differentiation in vitro, thereby maintaining a balanced generation of hematopoietic blood cells. Clustered retroviral therapeutic vector integrations into this genomic locus detected in the peripheral blood of patients several years after HSC gene therapy strongly hint towards expression and relevance of these genes in hematopoietic stem and progenitor cells. Previous studies focused either on EVL or miR-342 without considering their co-expression within the same locus.

On the molecular level, EVL was shown to play a pivotal role in actin cytoskeleton remodeling [26, 42] and homologous recombination. EVL is a member of the Ena/VASP family [43] and all three proteins are mainly involved in axon guidance, cell adhesion, and migration due to their actin-binding potential [44–47]. Homozygous *Evl* knockout mice are viable and fertile whereas the deletion of all three family members *Ena/Vasp/Evl* causes the development of brain defects and abnormal leukocyte migration [48]. Strongly in line with our results, showing the highest expression of EVL in B- and T-cell lineages, Lanier et al. detected a high expression of EVL in spleens and thymi of mice [44]. Moreover, we were able to show that the OE of EVL leads to the deregulation of pathways involved in B-cell and T-cell development and drives lymphopoiesis in vitro and in vivo. In contrast, miR-342 OE increases myeloid colony formation with a higher frequency of myelopoietic progenitors in vivo. In accordance with our data, recent studies demonstrated that miR-342 is a direct target of the transcription factor PU.1 leading to an accelerated all-trans retinoic acid-induced myeloid differentiation of APL blasts [49] and that miR-342 is expressed in human and murine macrophages playing an important role in pro-apoptotic signaling after IL-4 stimulation [50].

In contrast to the results of our study, it was suggested that miR-342 exerts a synergistic function with EVL [16] as it is co-expressed with its host gene [16, 51, 52]. Our subsequent investigation of promoter domains, active enhancer regions and transcription factor binding sites together with gene expression in hematopoietic cells revealed that miR-342 is indeed co-regulated with its host gene. This result is reinforced by the identification of miR-342 as a downstream target of Notch1 [53] suppressed via Hes5 binding sites within the *Evl* promoter [28]. We then tested the hypothesis whether increasing amounts of miR-342 influence the cellular localization of EVL, however, we have no indication for that (data not shown), which argues for an alteration of differentiation regulating expression programs. Global gene expression profiling of EVL^+^ or miR-342^+^ LSK cells strongly supports their molecular influence on lymphoid or myeloid lineage choice, respectively. We showed that simultaneous OE of both genes has a neutralizing effect on the hematopoietic lineage output. EVL abolished the myeloid-biased differentiation upon miR-342 expression, whereas the induction of miR-342 in EVL expressing LSK cells reduced lymphoid colony formation substantially. We demonstrate that the interaction of the host gene and its intronic miRNA is exerted by miR-342 mediated suppression of genes important in lymphopoiesis. In the same line, EVL driven miR-342 target genes may act as individual sponges, thereby suppressing the role of miR-342 in myelopoiesis, which is supported by a reduced myeloid colony output of miR-342^+^ LSK cells compared to mock transduced cells in presence of a specific sponge vector (data not shown). This clearly establishes the antagonistic role of miR-342 towards EVL driven lineage determination in hematopoiesis. Interestingly, Gao et al. [28] showed a modulatory role of miR-342 also on neural lineage differentiation in the brain. MiR-342 OE in neural stem cells promoted the generation of intermediate neural progenitors and inhibited the differentiation of astrocytes. Phylogenomic analyses describe the importance of actin-associated processes for phagocytosis and that genes involved in actin filament formation, actin binding or nucleation are highly conserved throughout eukaryotes [54]. Therefore, it is tempting to speculate that conserved gene loci involved in actin regulation like the *Evl/Mir342* locus have contributed to the development of innate and adaptive immune cells and are still relevant for their balanced generation.

We identified Tcf12 (Transcription Factor 12), a basic helix-loop-helix transcription factor, as one miR-342-targeted mRNA expressed in LSK cells. Its function is important for developmental processes like T-lymphopoiesis and neurogenesis [3, 55]. The disruption of the *Tcf12* locus in hESCs followed by mesodermal and hematopoietic differentiation in vitro clearly demonstrated the failure to generate committed T-cell precursors without an impact on myeloid differentiation [56]. This failure in early T-cell development could be fully restored by ectopic expression of HEBcan, the canonical protein transcribed from the *Tcf12* locus [56]. Remarkably, the deletion of HEB in mice led to a profound reduction of committed B-cells in vivo as well as in vitro due to a developmental defect within CLP [57], which is in line with the reduced B-cell colony formation upon OE of miR-342, further supporting that Tcf12 is a direct target of miR-342. Interestingly, TCF12 is also expressed in lymphoid malignancies [58], suggesting a role in B- and T-cell-derived leukemia. In addition, we identified Mitf and Irf8 as further miR-342 targets in hematopoietic cells. Importantly, Irf8 is significantly upregulated upon Evl OE in LSK cells, leading to the activation of downstream target molecules. In line with our data showing that miR-342, which targets Irf8, results in increased myeloid colony formation in vitro and in vivo, *Irf8* knockout mice exhibit expansion of granulocytes and macrophages [59], and Scheller et al. showed an increased sensitivity of *IRF8*^*−/−*^ myeloid progenitors to G-CSF and GM-CSF leading to myeloid expansion [60]. Our results strongly suggest that IRF8 plays a major role in balanced lineage output driven by *Evl/Mir342* via the IRF8/PU.1 axis. IRF8 has been shown to negatively regulate PU.1 [61], thereby controlling transcriptional programs for lymphoid or myeloid lineage specification by suppressing the other branch in return.

In summary, our data show that two hematopoietic differentiation programs are driven by one common and evolutionary highly conserved genetic locus. The interaction between EVL and miR-342 is important for hematopoietic differentiation by maintaining a balanced lineage output under steady-state conditions. This provides the blood system with a posttranscriptional mechanism to stabilize and quickly re-adjust lineage determination. Future studies are needed to show whether this is a common principle and whether additional genomic loci shared by host genes and intronic miRNAs have similar effects on balancing lineage determination also in other tissues and organs.

## Supplementary information

Supplementary Figure Legends

Supplementary Material

Supplementary Table S7

Supplementary Table S8

Figure S1

Figure S2

Figure S3

Figure S4

Figure S5

Figure S6
