# Supplementary material for: The balance between the intronic miR-342 and its host gene Evl determines hematopoietic cell fate decision
Source: Leukemia. 2021 May 21;35(10):2948–63. doi: 10.1038/s41375-021-01267-5 (PMC8478659; doi:10.1038/s41375-021-01267-5)
Supplement: Supplementary file 1 — Supplementary Figure Legends [file 41375_2021_1267_MOESM1_ESM.docx]

**Supplementary Figure Legends**

**Supplementary Figure 1:**

Sequence homology between murine and human EVL and co-expression of Evl and miR-342 in various cell lines.

(a.) Sequence homology between human and mouse EVL protein.

The top row (Query) shows the human EVL sequence while the bottom line (Sbjct) shows the mouse sequence. Sequence data was obtained from <https://www.ncbi.nlm.nih.gov/protein> and aligned using Protein Blast (blastp suite) from <https://blast.ncbi.nlm.nih.gov/Blast.cgi> using standard parameters. Vertical lines indicate the identical amino acids.

**(b.)** Relative expression (mean, SEM) of miR-342 (red bars) and Evl (green bars) in murine 32D and J774 cells.

**(c.)** Relative expression (mean, SEM) of miR-342 (red bars) and Evl (green bars) in human Jurkat and U2OS cells.

**Supplementary Figure 2:**

**Genetic features of the *EVL/MIR342* locus.**

Genomic localization of all therapeutic gamma-retroviral vector insertions (gRIS) close to the *EVL/MIR342* gene locus detected in 10 Wiskott-Aldrich-Syndrome (WAS1-10) gene therapy patients. Purple lines depict genomic interaction data (Hi-C sequencing results of human CD34^+^ cells; Mifsud et al. 2015) between promoter (pro) elements to other genomic regions (other). Genomic conservation of the *EVL/MIR342* genetic region from human (hg19) to mouse (mm9). Histone modifications in human CD34^+^ cells representing promoter (H3K4me3), enhancer (H3K4me1) and active (H3K27ac) chromatin regions as well as open chromatin detected by ATAC Sequencing in distinct murine hematopoietic cell populations (HSC to B cell). H - histone; K - Lysine; me - methylated; ac - acetylated; HSC - hematopoietic stem cell; MPP - multipotent progenitor; LMPP - lymphoid-primed multipotent progenitor; CMP - common myeloid progenitor; GMP - granulocyte macrophage progenitor; MEP - megakaryocytic erythroide progenitor; CLP - common lymphoid progenitor; NK - natural killer cell; HSPCs - hematopoietic stem and progenitor cells

**Supplementary Figure 3:**

**Stable and functional overexpression of Evl and miR-342 in hematopoietic cells.**

1. Scheme of lentiviral transfer vectors encoding for Evl, eGFP, miR-342 and mCherry. LTR - long terminal repeat; PGK - phosphoglycerate kinase promoter; UbiC - Ubiquitin C promoter; IRES - internal ribosomal entry site; eGFP - enhanced green fluorescent protein
2. Fold change of lentiviral-mediated Evl and miR-342 expression in 32D cells normalized to mock transduced (GFP or mCH) control cells five days after enrichment of eGFP and mCherry positive cells.
3. Western Blot validating EVL overexpression on protein level after LV transduction of 293T cells using the vectors LV.eGFP and LV.Evl.
4. Ratio of renilla/firefly luciferase intensity validating the specificity of overexpressed miR‑342 by binding to respective miRNA binding sites leading to luciferase suppression (** - P<.01).
5. Blue bars indicate decreased pathways upon Evl expression in murine primary LSK cells compared to mock transduced control cells evaluated using Ingenuity Pathway Analyzer, whereas orange bars represent activated pathways based on the activation z-score.

**Supplementary Figure 4:**

**Donor derived frequency of hematopoietic cells in spleens of transplanted mice.**

**(a.)** Frequency of donor-derived eGFP/mCherry positive cells in the spleen of primary (1°) and

**(b.)** secondary (2°) recipient mice after transplantation of LSK cells transduced with LV mediated vectors encoding for Evl, miR-342 or mock. Gr - granulocytes; MoMac - monocytes and macrophages, B - B cells; T - T cells

**Supplementary Figure 5:**

**Ectopic overexpression of either Evl or miR-342 has no influence on the partner transcript level.**

**(a.)** Relative expression (mean, SEM) of Evl (green bars) or miR-342-3p (red bars) in murine J774 cells, which are lentivirally transduced with a control vector (mCherry) or miR-342 overexpressing construct (miR-342), or with a control vector (eGFP) or Evl overexpressing construct (Evl).

(**b.)** Relative expression (mean, SEM) of Evl (green bars) or miR-343-3p (red bars) in human Jurkat and U2OS cells, which are lentivirally transduced with a control vector (mCherry) or miR-342 overexpressing construct (miR-342), or with a control vector (eGFP) or Evl overexpressing construct (Evl).

**Supplementary Figure 6:**

**Validation of miR-342-3p and miR-342-5p targets.**

**(a.)** Relative luciferase activity (normalized to gene specific mutated UTR vector transfected cells) after transfection with specific 3’UTR sequence containing constructs in 293T cells transfected with miR-342-3p mimic or

**(b.)** miR-342-5p mimic. *P*-values have been calculated using unpaired Students t-test 3’‑miR‑342 target sequence vs. mut sequence. * *p*<0.05; ** *p*<0.01; **** *p*<0.0001
