# Supplementary material for: The balance between the intronic miR-342 and its host gene Evl determines hematopoietic cell fate decision
Source: Leukemia. 2021 May 21;35(10):2948–63. doi: 10.1038/s41375-021-01267-5 (PMC8478659; doi:10.1038/s41375-021-01267-5)
Supplement: Supplementary file 2 — Supplementary Material [file 41375_2021_1267_MOESM2_ESM.docx]

**Supplementary Tables**

**Table S1: primers, oligos used for cloning**

| Gene | Forward primer | Reverse primer |
| --- | --- | --- |
| *MIR342*-locus | GCTAGCGGGTTCACCATGTGTTCTTCC | TGCTTGGTTTGGGGTGCTGCCGCGCGG |
| miR-342-binding sites | TCGAGACGGGTGCGATTTCTGTGTGAGAGATCACGGGTGCGATTTCTGTGTGAGAGC | GGCCGCTCTCACACAGAAATCGCACCCGTGATCTCTCACACAGAAATCGCACCCGTC |
| Tfdp2_UTR | TCGAGTGATGCTCATATGTGTCTTTAGTGTGAGCTCTTGTGTTTTGAAATGATTGCTTCAGTCTTTGCCTTTGTTTGCAGC | GGCCGCTGCAAACAAAGGCAAAGACTGAAGCAATCATTTCAAAACACAAGAGCTCACACTAAAGACACATATGAGCATCAC |
| Tfdp2_UTR_mut | TCGAGTGATGCTCATATGTGTCTTTAtttttttCTCTTGTGTTTTGAAATGATTGCTTCAGTCTTTGCCTTTGTTTGCAGC | GGCCGCTGCAAACAAAGGCAAAGACTGAAGCAATCATTTCAAAACACAAGAGtttttttCTAAAGACACATATGAGCATCAC |
| Tcf12_UTR | TCGAGAAACAGCAGTTGTTGCTATGATGTGTGAGTGAACATAAGCCACTGCCTGGCCTTTTTTCTTCAGAGCTTGTGC | GGCCGCACAAGCTCTGAAGAAAAAAGGCCAGGCAGTGGCTTATGTTCACTCACACATCATAGCAACAACTGCTGTTTC |
| Tcf12_UTR_mut | TCGAGAAACAGCAGTTGTTGCTATGATtttttttTGAACATAAGCCACTGCCTGGCCTTTTTTCTTCAGAGCTTGTGC | GGCCGCACAAGCTCTGAAGAAAAAAGGCCAGGCAGTGGCTTATGTTCAtttttttATCATAGCAACAACTGCTGTTTC |
| Mitf_UTR | TCGAGGTGAACTGATTTCTCCAAGTGTGAGCTTTCTGAGCAAGGGGATTTTTTTGCTTCAGAGAAGTAAGTGTCTGGC | GGCCGCCAGACACTTACTTCTCTGAAGCAAAAAAATCCCCTTGCTCAGAAAGCTCACACTTGGAGAAATCAGTTCACC |
| Mitf_UTR_mut | TCGAGGTGAACTGATTTCTCCAAtttttttCTTTCTGAGCAAGGGGATTTTTTTGCTTCAGAGAAGTAAGTGTCTGGC | GGCCGCCAGACACTTACTTCTCTGAAGCAAAAAAATCCCCTTGCTCAGAAAGtttttttTTGGA GAAATCAGTTCACC |
| Irf8_UTR | TCGAGGCCTCAGCCTCCTTAGCAAGGGGAGGAGAGGTGTGAGTCACTGTGAATCCTTAAAGGAATCTTTAAAATAAATCGC | GGCCGCGATTTATTTTAAAGATTCCTTTAAGGATTCACAGTGACTCACACCTCTCCTCCCCTTGCTAAGGAGGCTGAGGCC |
| Irf8_UTR_mut | TCGAGGCCTCAGCCTCCTTAGCAAGGGGAGGAGAGtttttttTCACTGTGAATCCTTAAAGGAATCTTTAAAATAAATCGC | GGCCGCGATTTATTTTAAAGATTCCTTTAAGGATTCACAGTGAtttttttCTCTCCTCCCCTTGCTAAGGAGGCTGAGGCC |
| Casp9_UTR | TCGAGGCCAGCTGCCAGGAGCACCCCTCCTGAGATCTGCCAACCAAAAATGTCTTCAGACATTGTCACATGTGCCCTGGGC | GGCCGCCCAGGGCACATGTGACAATGTCTGAAGACATTTTTGGTTGGCAGATCTCAGGAGGGGTGCTCCTGGCAGCTGGCC |
| Casp9_UTR_mut | TCGAGGCCAGCTGCCAGGAtttttttTCCTGAGATCTGCCAACCAAAAATGTCTTCAGACATTGTCACATGTGCCCTGGGC | GGCCGCCCAGGGCACATGTGACAATGTCTGAAGACATTTTTGGTTGGCAGATCTCAGGAtttttttTCCTGGCAGCTGGCC |
| Limk1_UTR | TCGAGGAGGGAAGGAGGCAGCCACCCACCAGCACCCCAGCCCTCCAGGGTCATCTCAGCACCCC | GGCCGGGGTGCTGAGATGACCCTGGAGGGCTGGGGTGCTGGTGGGTGGCTGCCTCCTTCCCTCC |
| Limk1_UTR_mut | TCGAGGAGGGAAGGAGGCAGCCACCCACCAttttttttGCCCTCCAGGGTCATCTCAGCACCCC | GGCCGGGGTGCTGAGATGACCCTGGAGGGCttttttttTGGTGGGTGGCTGCCTCCTTCCCTCC |
| Phc1_UTR | TCGAGGTGACACATGTGGCTGTTGCCATTCTTTCTGCACCCCAGCTCCCAACTGTTCCCACCTTCAACCCTCTC | GGCCGAGAGGGTTGAAGGTGGGAACAGTTGGGAGCTGGGGTGCAGAAAGAATGGCAACAGCCACATGTGTCACC |
| Phc1_UTR_mut | TCGAGGTGACACATGTGGCTGTTGCCATTCTTTCTttttttttGCTCCCAACTGTTCCCACCTTCAACCCTCTC | GGCCGAGAGGGTTGAAGGTGGGAACAGTTGGGAGCttttttttAGAAAGAATGGCAACAGCCACATGTGTCACC |

**Table S2: TaqMan Assays used for qRT-PCR**

| Gene | Manufacturer | Cat. No. | Assay ID |
| --- | --- | --- | --- |
| Evl | ThermoFisher Scientific | #4448892 | Mm00468405_m1 |
| Gapdh | ThermoFisher Scientific | #4331182 | Mm99999915_g1 |

**Table S3: Primers used for qRT-PCR based on SYBR Green**

| Gene | Forward primer | Reverse primer |
| --- | --- | --- |
| Evl_cod | ATCTACCACAACACCGCCAG | AGGTGGTGGCTTCCTCTTTG |
| Tbp | CCTTCACCAATGACTCCTATGAC | CAAGTTTACAGCCAAGATTCAC |
| miR-342 | TCTCACACAGAAATCGCACCCGT | miScript SYBR^®^ Green PCR Kit |
| Rnu6b | ACACGCAAATTCGTGAAGCGCT | miScript SYBR^®^ Green PCR Kit |

**Table S4: Primary and Secondary Antibodies used for Western Blot or Immunprecipitation**

| Antibody | Host | Manufacturer | Order number | Dilution |
| --- | --- | --- | --- | --- |
| anti-EVL | goat | Santa Cruz | sc-66527 | 1:1 000 |
| anti-EVL | mouse | Santa Cruz | sc-373793 | 1:1 000 |
| anti-PHC1 | rabbit | Cell Signaling | 13505 | 1:1 000 |
| anti-LIMK1 | rabbit | Cell Signaling | 3842 | 1:1 000 |
| anti-CASP9 | rabbit | Cell Signaling | 9504 | 1:1 000 |
| anti-βActin-HRP |  | Sigma |  | 1:10 000 |
| anti-Ago2 | mouse | Wako Chemicals | 292-67301 | 5 µg/reaction |
| IgG isotype | mouse | Sigma Aldrich | I5381 | 5 µg/reaction |
| anti-goat-HRP | Rabbit | Abcam | ab6741 | 1:10 000 |
| anti-mouse-HRP | Rabbit | Abcam | ab6728 | 1:10 000 |
| anti-rabbit-HRP | Goat | Abcam | ab6721 | 1:10 000 |

**Table S5: Immunophenotypic definition of hematopoietic cell populations**

| Abbreviation | Cell type | source | Immunphenotypic definition |
| --- | --- | --- | --- |
| LSK | Hematopoietic stem and progenitor cells | BM | Lineage^-^, CD117^+^, Ly6A/E^+^ |
| LT-HSC | Long-term hematopoietic stem cells | BM | CD34^-^, CD150^+^, CD48^-^, CD135^-^, Lineage^-^, CD117^+^, Ly6A/E^+^ |
| ST-HSC/MPP1 | Short-term hematopoietic stem cells, Multipotent progenitors 1 | BM | CD34^+^, CD150^+^, CD48^-^, CD135^-^, Lineage^-^, CD117^+^, Ly6A/E^+^ |
| MPP2 | Multipotent progenitors 2 | BM | CD34^+^, CD150^+^, CD48^+^, CD135^-^, Lineage^-^, CD117^+^, Ly6A/E^+^ |
| MPP3 | Multipotent progenitors 3 | BM | CD34^+^, CD150^-^, CD48^+^, CD135^-^, Lineage^-^, CD117^+^, Ly6A/E^+^ |
| MPP4 | Multipotent progenitors 4 | BM | CD34^+^, CD150^-^, CD48^+^, CD135^+^, Lineage^-^, CD117^+^, Ly6A/E^+^ |
| CMP | Common myeloid progenitor | BM | Lineage^-^, CD117^+^, Ly6A/E^-^, CD34^+^, CD16/32^int^ |
| GMP | Granulocyte macrophage progenitor | BM | Lineage^-^, CD117^+^, Ly6A/E^-^, CD34^+^, CD16/32^+^ |
| MEP | Megakaryocyte erythroid progenitors | BM | Lineage^-^, CD117^+^, Ly6A/E^-^, CD34^-^, CD16/32^-^ |
| CLP | Common lymphoid progenitors | BM | Lineage^-^, CD117^int^, Ly6A/E^int^, CD127^+^ |
| Gr | Granulocytes | PB | CD11b^+^, Ly6G^+^ |
| Macro/Mono/DC | Macrophages, Monocytes, Dendritic cells | PB | CD11b^+^, Ly6G^-^ |
| Ery | Erythroid progenitor cells | PB | Ter119^+^ |
| T cells | T cells | PB | CD3^+^ |
| CD4 | CD4 positive T cells (mature T helper cells) | PB | CD3^+^, CD4^+^ |
| CD8 | CD8 positive T cells (cytotoxic T cells) | PB | CD3^+^, CD8^+^ |
| B cells | B cells | PB | CD45R^+^ |

**Tables S6: Antibodies used for flow cytometry**

Sort of LT-HSC & MPP populations

| Antibody | Manufacturer | Conjugate | Order number | Dilution |
| --- | --- | --- | --- | --- |
| CD117 | BD | APC | 553991 | 1:200 |
| CD135 | BD | PE | 553930 | 1:100 |
| CD150 | BD | PE-Cy5 | 555276 | 1:500 |
| CD34 | BD | FITC | 560238 | 1:30 |
| CD48 | Biozol | AlexaFlour700 | B188338 | 1:400 |
| Lineage Cocktail | See below | PE-Cy7 | See below | 1:400 |
| Ly-6A/E  (Sca-1) | BD | APC-Cy7 | 552770 | 1:200 |

Sort of Progenitors

| Antibody | Manufacturer | Conjugate | Order number | Dilution |
| --- | --- | --- | --- | --- |
| CD117 | BD | APC | 553991 | 1:200 |
| CD127 (IL7R) | eBioscience | PE-Cy5 | 15-1271-82 | 1:200 |
| CD16/32 (FcγR) | BioLegend | PE | 101308 | 1:200 |
| CD34 | BD | FITC | 560238 | 1:30 |
| Lineage Cocktail | See below | PE-Cy7 | See below | 1:300 |
| Ly-6A/E (Sca-1) | BD | APC-Cy7 | 552770 | 1:200 |

Lineage Cocktail PE-Cy7

| Antibody | Manufacturer | Conjugate | Order number | Dilution |
| --- | --- | --- | --- | --- |
| CD11b | BD | PE-Cy7 | 552850 | 1:400 |
| CD3 | BD | PE-Cy7 | 552849 | 1:400 |
| CD45R | BD | PE-Cy7 | 552772 | 1:400 |
| Ly6G/C (Gr-1) | BD | PE-Cy7 | 552894 | 1:400 |
| Ter119 | BD | PE-Cy7 | 553673 | 1:400 |

Sort of LSK cell population

| Antibody | Manufacturer | Conjugate | Order number | Dilution |
| --- | --- | --- | --- | --- |
| Lineage cocktail | BD | APC | 558074 | 1:100 |
| Ly6A/E (Sca1) | BD | PE-Cy7 | 558162 | 1:200 |
| CD117 (cKit) | BD | PE | 553355 | 1:200 |

BM Analysis of transplanted mice (CD45.1) - 1

| Antibody | Manufacturer | Conjugate | Order number | Dilution |
| --- | --- | --- | --- | --- |
| CD117 | BD | PE | 553355 | 1:200 |
| CD150 | BD | PE-Cy5 | 555276 | 1:500 |
| CD34 | BD | AlexaFlour700 | 560518 | 1:30 |
| CD45.1 | BioLegend | PacificBlue | 110722 | 1:100 |
| CD48 | BD | PE-Cy7 | 560731 | 1:200 |
| Lineage Cocktail | BD | APC | 558074 | 1:100 |
| Ly6A/E (Sca1) | BD | APC-Cy7 | 552770 | 1:200 |

BM Analysis of transplanted mice (CD45.1) - 2

| Antibody | Manufacturer | Conjugate | Order number | Dilution |
| --- | --- | --- | --- | --- |
| CD117 | BD | PE-Cy7 | 558163 | 1:200 |
| CD127 (IL7R) | eBioscience | PE-Cy5 | 15-1271-82 | 1:300 |
| CD16/32 (FcγR) | BioLegend | PE | 101308 | 1:200 |
| CD34 | BD | AlexaFlour700 | 560518 | 1:30 |
| CD45.1 | BioLegend | PacificBlue | 110722 | 1:100 |
| Lineage Cocktail | BD | APC | 558074 | 1:100 |
| Ly6A/E (Sca1) | BD | APC-Cy7 | 552770 | 1:200 |

PB and Spleen Analysis of transplanted mice

| Antibody | Manufacturer | Conjugate | Order number | Dilution |
| --- | --- | --- | --- | --- |
| CD11b | BD | PerCP-Cy5.5 | 550764 | 1:200 |
| CD3 | BD | PerCP-Cy5.5 | 560527 | 1:200 |
| CD45.1 | BD | PE | 553930 | 1:200 |
| CD45.2 | BD | APC | 558702 | 1:200 |
| CD45R | BD | AlexaFluor700 | 557957 | 1:200 |
| Ly6G | BD | AlexaFluor700 | 561236 | 1:200 |

**Supplementary Methods**

**Global Gene Expression Profiling**

Global gene expression profiling was conducted with the Illumina MouseWG-6v2_BeadChip system. After applying a ≥2-fold change as threshold, the data was analyzed using the Ingenuity Pathway Analyzer (Qiagen) and DAVID (1, 2). The data discussed in this publication have been deposited in NCBI's Gene Expression Omnibus (3). RNA Seq data of 13 primary cell types were downloaded from Corces et al., 2016 (4). RNA-Seq raw counts were converted to transcripts per million (TPM) using a custom python script and normalized using the R package DESeq2 (5).

We re-analyzed the publicly available gene expression data set of 2 096 leukemia samples of the MILE study (6) (Microarray Innovations in LEukemia) from the ELN (European Leukemia Network) to evaluate the expression of Evl in distinct leukemia subtypes versus healthy controls.

SmallRNA Seq data of 14 AML patients and 3 healthy donors were provided by Krakowsky et al., 2018 (7) and normalized using the R package DESeq2. Expression analyses, clinical features and molecular associations of the precursor mir-342 in AML patients were analyzed using „The Cancer Genome Atlas” (TCGA) database (8). We used a recently published data set (9) to assess the physical genetic interaction of the *EVL/MIR342* locus determined by promoter capture HiC sequencing.

**Low RNA high-throughput sequencing after Ago2-IP and bioinformatical analysis**

Total RNA fractions were submitted for low RNA HTS using Illumina HiSeq2000 (paired end, 125bp). The reads were first subjected to adapter clipping, and filtering out poly(A) tails, artefacts and reads shorter than 17bp. Then, they were mapped with STAR (10) against the mouse genome GRCm38 (73% uniquely mapped reads, 25% multiple mapped reads). After using htseq-count (11) with gencode.vM8.annotation.gtf (12) and the unstranded parameter, the counts of the samples were normalized to counts per million and fold changes were calculated (custom perl script).

The common predicted miR-342 target genes (from miRWalk, miRanda, RNA22 and Targetscan) were downloaded from miRWalk2.0 (13) and used in BioVenn (14) to identify the intersection between genes expressed in LSK cells (RPKM>800) (15) and the enriched genes after AGO2 pulldown.

**Supplementary References**

1. Huang da W, Sherman BT, Lempicki RA. Systematic and integrative analysis of large gene lists using DAVID bioinformatics resources. Nat Protoc. 2009;4(1):44-57.

2. Huang da W, Sherman BT, Lempicki RA. Bioinformatics enrichment tools: paths toward the comprehensive functional analysis of large gene lists. Nucleic Acids Res. 2009;37(1):1-13.

3. Edgar R, Domrachev M, Lash AE. Gene Expression Omnibus: NCBI gene expression and hybridization array data repository. Nucleic Acids Res. 2002;30(1):207-10.

4. Corces MR, Buenrostro JD, Wu B, Greenside PG, Chan SM, Koenig JL, et al. Lineage-specific and single-cell chromatin accessibility charts human hematopoiesis and leukemia evolution. Nat Genet. 2016;48(10):1193-203.

5. Love MI, Huber W, Anders S. Moderated estimation of fold change and dispersion for RNA-seq data with DESeq2. Genome Biol. 2014;15(12):550.

6. Haferlach T, Kohlmann A, Wieczorek L, Basso G, Kronnie GT, Bene MC, et al. Clinical utility of microarray-based gene expression profiling in the diagnosis and subclassification of leukemia: report from the International Microarray Innovations in Leukemia Study Group. J Clin Oncol. 2010;28(15):2529-37.

7. Krakowsky RHE, Wurm AA, Gerloff D, Katzerke C, Brauer-Hartmann D, Hartmann JU, et al. miR-451a abrogates treatment resistance in FLT3-ITD-positive acute myeloid leukemia. Blood Cancer J. 2018;8(3):36.

8. Cancer Genome Atlas Research N, Ley TJ, Miller C, Ding L, Raphael BJ, Mungall AJ, et al. Genomic and epigenomic landscapes of adult de novo acute myeloid leukemia. N Engl J Med. 2013;368(22):2059-74.

9. Mifsud B, Tavares-Cadete F, Young AN, Sugar R, Schoenfelder S, Ferreira L, et al. Mapping long-range promoter contacts in human cells with high-resolution capture Hi-C. Nat Genet. 2015;47(6):598-606.

10. Dobin A, Davis CA, Schlesinger F, Drenkow J, Zaleski C, Jha S, et al. STAR: ultrafast universal RNA-seq aligner. Bioinformatics. 2013;29(1):15-21.

11. Anders S, Pyl PT, Huber W. HTSeq--a Python framework to work with high-throughput sequencing data. Bioinformatics. 2015;31(2):166-9.

12. Mudge JM, Harrow J. Creating reference gene annotation for the mouse C57BL6/J genome assembly. Mamm Genome. 2015;26(9-10):366-78.

13. Dweep H, Gretz N. miRWalk2.0: a comprehensive atlas of microRNA-target interactions. Nat Methods. 2015;12(8):697.

14. Hulsen T, de Vlieg J, Alkema W. BioVenn - a web application for the comparison and visualization of biological lists using area-proportional Venn diagrams. BMC Genomics. 2008;9:488.

15. Cabezas-Wallscheid N, Klimmeck D, Hansson J, Lipka DB, Reyes A, Wang Q, et al. Identification of regulatory networks in HSCs and their immediate progeny via integrated proteome, transcriptome, and DNA methylome analysis. Cell Stem Cell. 2014;15(4):507-22.
