# Supplementary material for: The balance between the intronic miR-342 and its host gene Evl determines hematopoietic cell fate decision
Source: Leukemia. 2021 May 21;35(10):2948–63. doi: 10.1038/s41375-021-01267-5 (PMC8478659; doi:10.1038/s41375-021-01267-5)
Supplement: Supplementary file 4 — Supplementary Table S8 [file 41375_2021_1267_MOESM4_ESM.docx]

**Supplementary Table S8**

**Table S8: Overview of the correlation between mir-342 expression and clinical, molecular features in AML (TCGA-LAML; n=188).** OS - overall survival; EFS - event free survival; FAB - French-American-British classification of AML

|  | Low mir-342 expression | High mir-342 expression | *P*-value |
| --- | --- | --- | --- |
| n | 94 | 94 |  |
| OS (median) range | 731 days (0-2284) | 485 days (0-2861) | 0.69 |
| EFS (median) range | 31.6 months (0-100.5) | 16.1 months (0-99.9) | 0.11 |
| Bone marrow blasts (mean) | 74.93 | 64.05 | **<0.0001***** |
| Peripheral blood blasts (mean) | 48.78 | 30.28 | **<0.0001***** |
| FAB subtype  M0  M1  M2  M3  M4  M5  M6  M7 | (n=93)  4  25  16  7  26  15  0  0 | (n=93)  13  20  25  10  14  6  2  3 | **0.04***  0.49  0.16  0.61  **0.049***  0.06  0.50  0.25 |
| Risk group  Favorable  Intermediate  Adverse | 18  61  13 | 17  46  30 | 0.85  **0.03***  **0.005**** |
| *FLT3 ITD* | 24 | 14 | 0.10 |
| *NPM1* | 45 | 7 | **<0.0001***** |
| *DNMT3A* | 31 | 15 | **0.01*** |
| *IDH1/2* | 22 | 13 | 0.13 |
| *RUNX1* | 3 | 14 | **0.009**** |
| *TP53* | 1 | 14 | **0.0006***** |
