# Supplementary material for: The balance between the intronic miR-342 and its host gene Evl determines hematopoietic cell fate decision
Source: Leukemia. 2021 May 21;35(10):2948–63. doi: 10.1038/s41375-021-01267-5 (PMC8478659; doi:10.1038/s41375-021-01267-5)

Supplementary Figure 1

a.

Score	Expect	Method	Identities	Positives	Gaps
670 bits(1729)	0.0	Compositional matrix adjust.	391/416(94%)	405/416(97%)	2/416(0%)

```
Query  1    MSEQSICQARASVMVYDDTSKKWVPIKPGQQGFSRINIYHNTASNTFRVVGVKLQDQQVV  60
            MSEQSICQARASVMVYDDTSKKWVPIKPGQQGFSRINIYHNTAS+TFRVVGVKLQDQQVV
Sbjct  1    MSEQSICQARASVMVYDDTSKKWVPIKPGQQGFSRINIYHNTASSTFRVVGVKLQDQQVV  60

Query  61   INYSIVKGLKYNQATPTFHQWRDARQVYGLNFASKEEATTFSNAMLFALNIMNSQEGGPS  120
            INYSIVKGLKYNQATPTFHQWRDARQVYGLNFASKEEATTFSNAMLFALNIMNSQEGGPS
Sbjct  61   INYSIVKGLKYNQATPTFHQWRDARQVYGLNFASKEEATTFSNAMLFALNIMNSQEGGPS  120

Query  121  SQRQVQNGPSPDEMDIQRRQVMEQHQQQRQESLERRTSATGPILPPGHPSSAASAPVSCS  180
            +QRQVQNGPSP+EMDIQRRQVMEQ  Q RQESLERR SATGPILPPGHPSSAAS   +SCS
Sbjct  121  TQRQVQNGPSPEEMDIQRRQVMEQ--QHRQESLERRISATGPILPPGHPSSAASTTLSCS  178

Query  181  GPPPPPPPPVPPPPTGATPPPPPPLPAGGAQGSSHDESSMSGLAAAIAGAKLRRVQRPED  240
            GPPPPPPPPVPPPPTG+TPPPPPPLPAGGAQG++HDESS SGLAAA+AGAKLRRVQRPED
Sbjct  179  GPPPPPPPPVPPPPTGSTPPPPPPLPAGGAQGTNHDESSASGLAAALAGAKLRRVQRPED  238

Query  241  ASGGSSPSGTSKSDANRASSGGGGGGLMEEMNKLLAKRRKAASQSDKPAEKKEDESQMED  300
            ASGGSSPSGTSKSDANRASSGGGGGGLMEEMNKLLAKRRKAASQ+DKPA++KEDESQ ED
Sbjct  239  ASGGSSPSGTSKSDANRASSGGGGGGLMEEMNKLLAKRRKAASQTDKPADRKEDESQTED  298

Query  301  PSTSPSPGTRAASQPPNSSEAGRKPWERSNSVEKPVSSILSRTPSVAKSPEAKSPLQSQP  360
            PSTSPSPGTRA SQPPNSSEAGRKPWERSNSVEKPVSS+LSRTPSVAKSPEAKSPLQSQP
Sbjct  299  PSTSPSPGTRATSQPPNSSEAGRKPWERSNSVEKPVSSLLSRTPSVAKSPEAKSPLQSQP  358

Query  361  HSRMKPAGSVNDMALDAFDLDRMKQEILEEVVRELHKVKEEIIDAIRQELSGISTT  416
            HSR+KPAGSVND+ LDA DLDRMKQEILEEVVRELHKVKEEIIDAIRQELSGISTT
Sbjct  359  HSRVKPAGSVNDVGLDALDLDRMKQEILEEVVRELHKVKEEIIDAIRQELSGISTT  414
```

b.

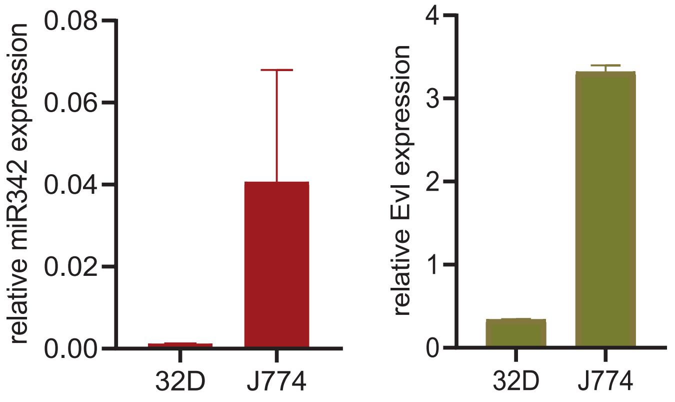

c.

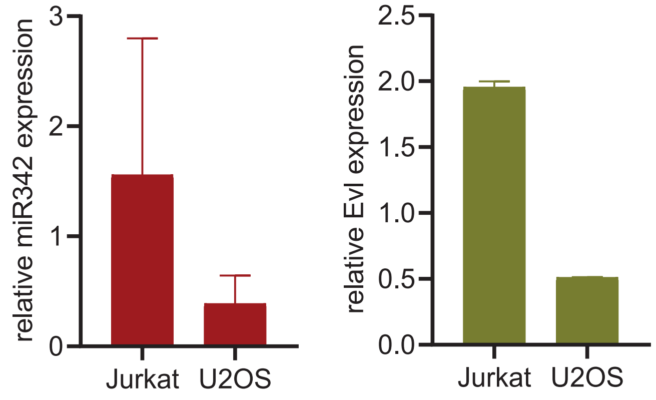

Supplement: Supplementary file 5 — Figure S1 [file 41375_2021_1267_MOESM5_ESM.pdf]
