# Supplementary figures and images for: The balance between the intronic miR-342 and its host gene Evl determines hematopoietic cell fate decision
Source: Leukemia. 2021 May 21;35(10):2948–63. doi: 10.1038/s41375-021-01267-5 (PMC8478659; doi:10.1038/s41375-021-01267-5)

Supplementary Figure 2

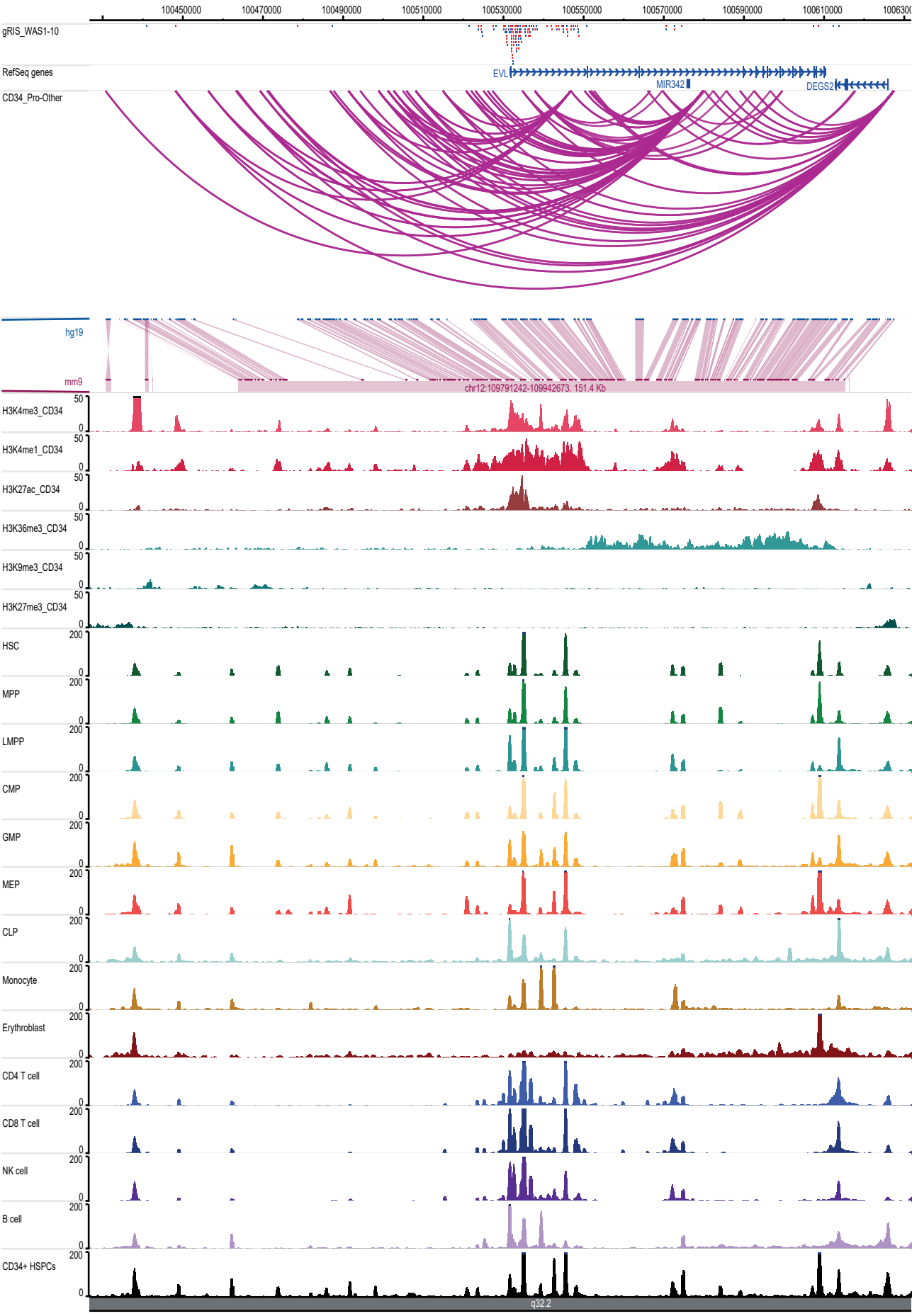

Supplement: Supplementary file 6 — Figure S2 [file 41375_2021_1267_MOESM6_ESM.pdf]

Supplementary Figure 3

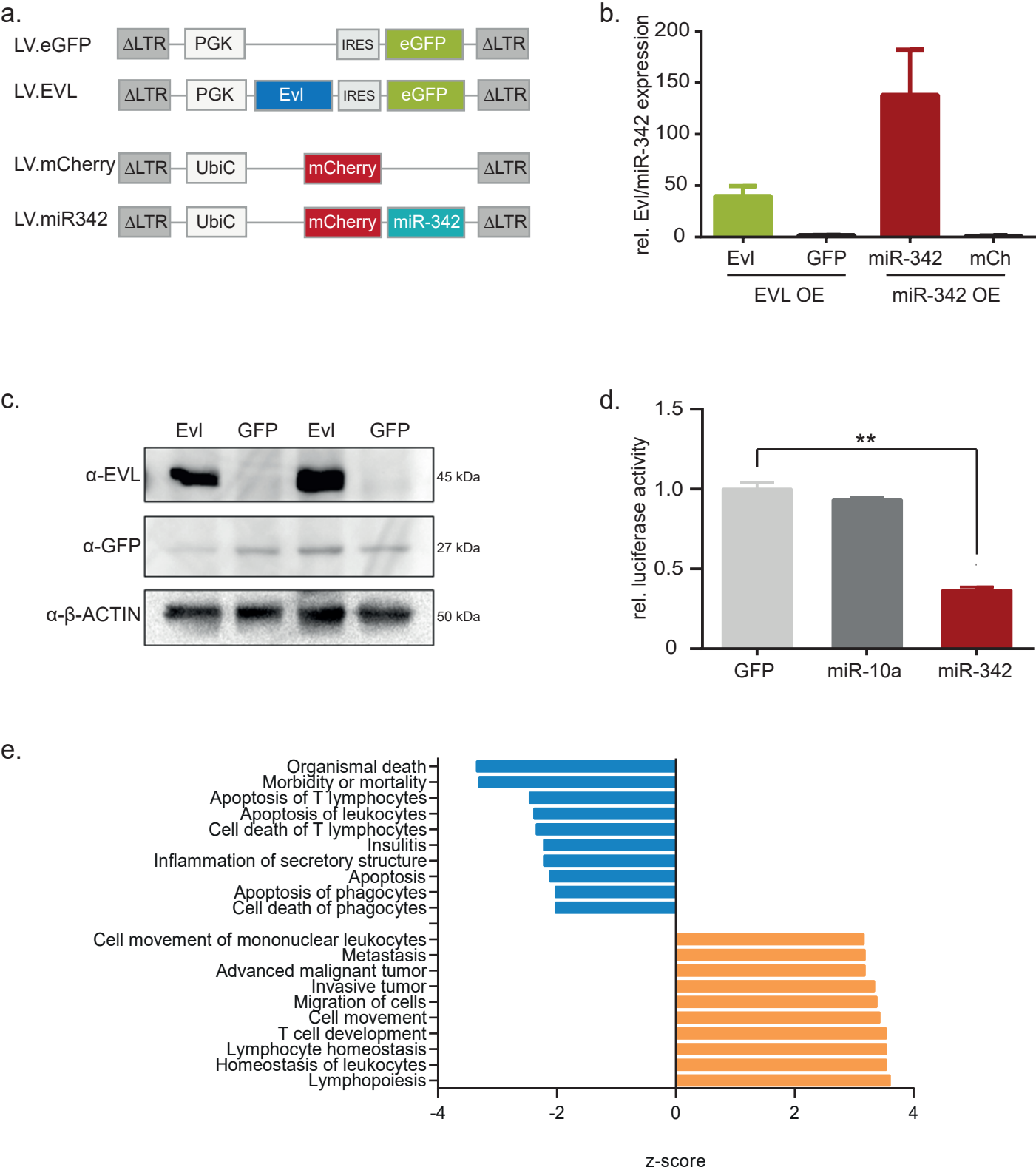

Supplement: Supplementary file 7 — Figure S3 [file 41375_2021_1267_MOESM7_ESM.pdf]

Supplementary Figure 4

a.

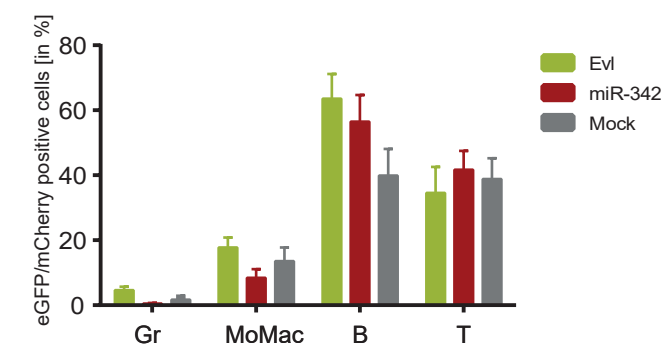

b.

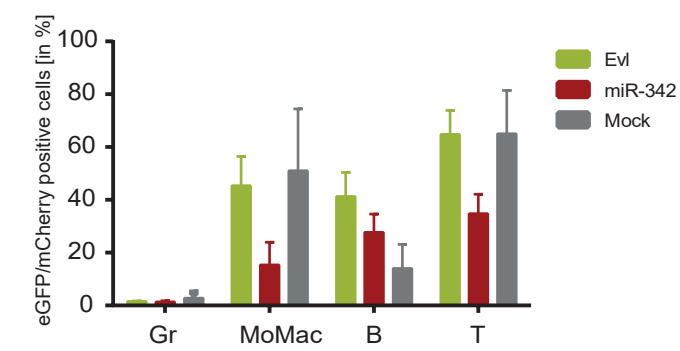

Supplement: Supplementary file 8 — Figure S4 [file 41375_2021_1267_MOESM8_ESM.pdf]

Supplementary Figure 5

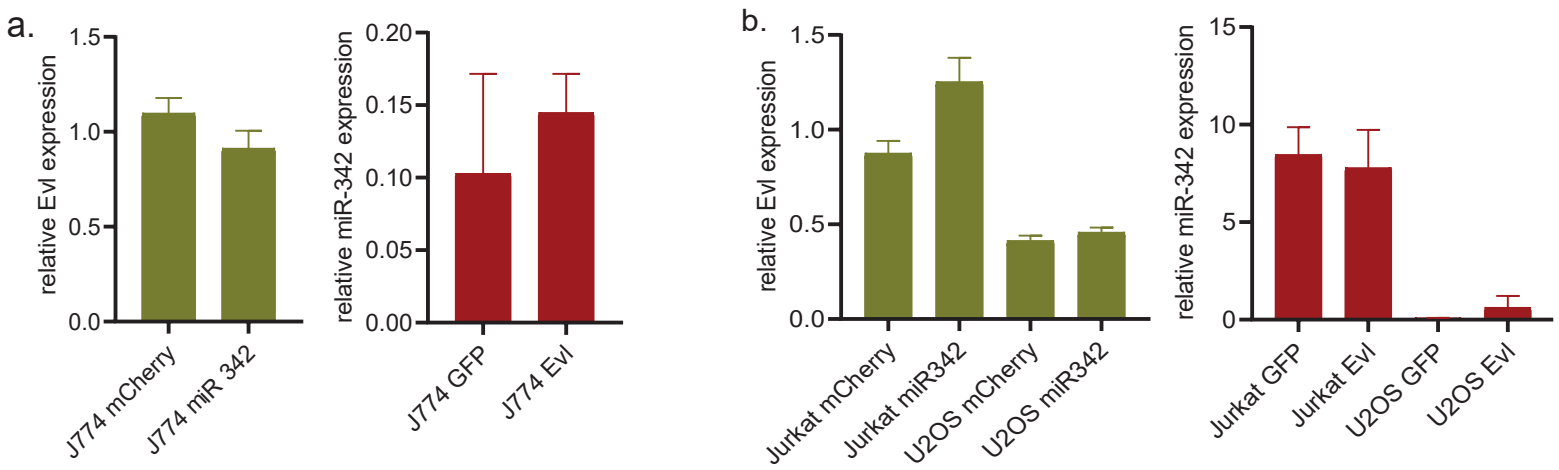

Supplement: Supplementary file 9 — Figure S5 [file 41375_2021_1267_MOESM9_ESM.pdf]

Supplementary Figure 6

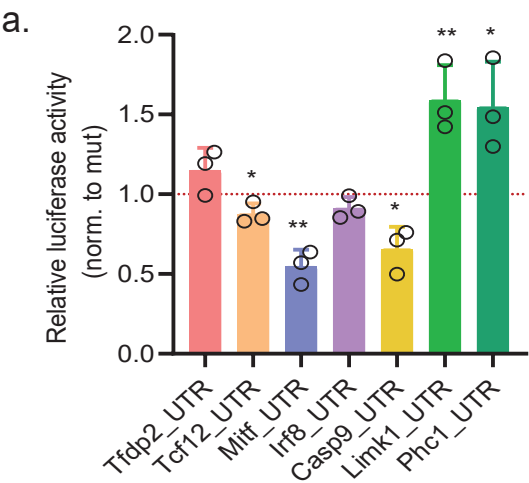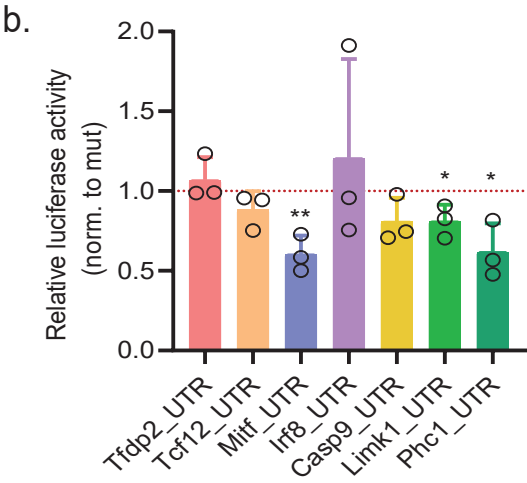

Supplement: Supplementary file 10 — Figure S6 [file 41375_2021_1267_MOESM10_ESM.pdf]
